# Book Notices

**Published:** 1878-09-20

**Authors:** 


### BOOK NOTICES.

ANATOMY DESCRIPTIVE AND SURGICAL, by Henry Gray, F. R. S. Fellow of the Royal College of Surgeons and Lecturer on Anatomy at St. George's Hospital Medical School, with five hundred and twenty-two engravings on wood. The drawings by H. V. Carter, M.D., and Dr. Westmacott. The dissections jointly by the author and Dr. Carter, with an introduction on General Anatomy and Development, by T. Holmes, A. Cantab, Surg. to St. George's Hospital, etc. A new American from the eighth and enlar ged English edition. To which is added "Lan marks, Medical and Surgical'' by Luther Holden, F. R. C. S., Surgeon to St. Bartholomew, etc., etc. Phiiadelphia: Henry C. Lea, 1878.

This excellent work on Anatomy is now so well known and so popular, both in Europe and America that any extensive remarks in regard to its merits would seem to be entirely unnecessary. The copy before us is the 8th and latest edition—an elegantly bound, compact volume of 983 pages. As in former American reprints, it has passed through the press under the supervision of Dr. Richard J. Dunglison. The work had already been three times revised by the distinguished editor, Mr. Holmes, since the last American edition, and now comes to us carefully corrected of every error that had escaped attention in England. Holden's *Landmarks, Medical and Surgical* have been appended in the present volume, which adds to its interest both for the student and general practitioner. It is extensively illustrated, and splendid in typographical taste and arrangement.

---

FOWN'S MANUAL OF CHEMISTRY—Theoretical and Practical, revised and corrected by Henry Watts, B.A., F. R. S., editor of the Journal of the Chemical Society, author of a "Dictionary of Chemistry," etc. A new American from the twelfth English edition, edited by Robert Bridges, M.D., Prof. of Chemistry in Philadelphia College of Pharmacy, with one hundred and seventy-seven illustrations. Philadelphia: Henry C. Lea.

This excellent work on Chemistry has been greatly enlarged and improved. Its typographical execution is fine, the type smaller but clear and distinct, and the volume sufficiently full for most purposes, containing over 1,000 pages. The internal arrangement of the work is admirable, and contains all the late discoveries and improvements in Cheistry. We hesitate not to recommend this work to both student and practitioner, and as well adapted also for use in our high schools and colleges.

---

DETERIORATICN AND RACE EDUCATION, with practical application to the condition of the people and industry, by Samuel Royce. Boston, Lee & Shepherd, Publishers; New York, Charles T. Dillingham, 1878.

This is a very readable work of 585 pages, dedicated to Mrs. Elizabeth Thompson, "the patriot and philanthropist, who devotes her energies to the elevation of the masses through industrial education, and labors for the improvement of the character of the men and women of America," etc.

Certainly no more important subject could engage the attention of the intelligent reader than the elevation of the masses of mankind. Whether viewed simply in its bearing upon our civil polity as a people, or from a moral and religious standpoint, this work cannot fail to attract the interest of the enlightened reader. We bespeak for it a God-speed and a world-wide circulation.

---

TRANSACTIONS of the American Gynæcological Society, vol. 11, for the year 1877.

This is a most valuable and instructive volume, containing articles of interest, too numerous to mention here. In addition, it contains an index to the gynæcological and obstetrical literature of all countries, from July '76 to January, '77. Price of vol. 11, $5.50. For the two vols. of '76 and '77, $10. Address Houghton Osgood & Co., Boston.

---

TRANSACTIONS OF THE AMERICAN MEDICAL ASSOCIATION, vol. xxviii., Philadelphia; Collins, Printer, 705 Jayne st.

This is a valuable number of The Transactions. It is a volume of 694 pages, containing many able and elaborate papers, and matter both instructive and interesting to the Practitioner.

---

THE reference and dose book, by C. Henri Leonard, M. A., M. D. Third edition, revised and enlarged. Detroit, S. E. corner Gratiot and Woodward Avenues, 1877.

A useful little work, containing in addition to doses, incompatibles, Jests for urine, viscoral measurements, pronunciation of names, etc.

---

WARNER & COMPANY.

The following very high testimonial, being a cable message from the Great Paris Exposition, was recently received by Wm. R. Warner & Co., Philadelphia;

*To Warner*, 1228 *Market, Philadelphia:*
*Awarded highest given pills; bronze medal.*
*St, Martin*, 11 *Paris*, (*Go. C.*, 2:54 *p. m.*)
*Received at Phiadelphia, August* 31*st*, 1878.

---

GLYCERITE OF KEPHALINE.

See new advertisement of the above article. Prepared by Dr. C. G. Polk of Philadelphia, and sold by Albert C. Dung, of New York.

---

☞ REMITTANCES are exceedingly short. Our friends do not heed onr appeals, nor consider our necessities. Strange!

